# Pipe(line) dreams for MicroED/3DED sample preparation

**DOI:** 10.1063/4.0001005

**Published:** 2025-10-27

**Authors:** Katherine A Spoth, Gabrielle R Budziszewski, M Elizabeth Snell, Miranda L Lynch, Devrim Acehan, Sarah EJ Bowman

**Affiliations:** 1 UB HWI, Buffalo, NY, USA; 2 University at Buffalo, Jacobs School of Medicine and Biomedical Science, Department of Structural Biology, Buffalo, NY, USA; 3 University at Buffalo, Jacobs School of Medicine and Biomedical Science, Department of Biochemistry, Buffalo, NY, USA

## Abstract

Microcrystal electron diffraction (MicroED) has experienced incredible growth as a powerful technique for structure determination. Similar to all diffraction-based structural methods for biomolecules, a bottleneck in using MicroED to solve structures is the need to generate crystals. The challenge of making crystals is slightly different for MicroED, since the crystals required are in the submicron size range, smaller than the wavelength of visible light and therefore difficult to detect. Given this requirement, a host of methods for generating crystals in the correct size regime for MicroED have emerged, including techniques for processing macrocrystals into the appropriate size range (via crushing, vortexing, FIB milling, etc) to direct growth and detection of submicron-sized crystals. Here we will describe our efforts in developing pipelines making use of our crystallization expertise to make nanocrystals for MicroED.

